# Editorial: Disorders of sex development in children: advancing multidisciplinary approaches for complex diagnosis and management

**DOI:** 10.3389/fped.2025.1646197

**Published:** 2025-07-01

**Authors:** Saida Hidouri, Evangelia Kalaitzoglou, Christopher J. Romero

**Affiliations:** ^1^ Department of Pediatric Surgery, Zaghouan Hospital, Zghoaun, Faculty of Medicine of Monastir, LR12SP13, Monastir, Tunisia; ^2^ Department of Pediatrics and Barnstable Brown Diabetes Center, University of Kentucky, Lexington, KY, United States; ^3^ Icahn School of Medicine at Mount Sinai Hospital, New York, NY, United States

**Keywords:** disorders of sexual development (DSD), chromosomal aneuploidies, sexual differentiation, gonadal abnormalities, genetic evaluation, gonadal tumors

**Editorial on the Research Topic**
Disorders of sex development in children: advancing multidisciplinary approaches for complex diagnosis and management

The term Differences of Sex Development (DSD), previously known as Disorders of Sex Development refers to a heterogeneous spectrum of congenital conditions in which the development of chromosomal, gonadal, or anatomic sex is not typically male or female (1). Investigative studies have shown that the presence, absence, or duplication of either sex chromosomes (X or Y) and various gene abnormalities required for gonadal development can profoundly impact the phenotype of the fetus (2). DSDs pose complex clinical challenges, requiring multidisciplinary approaches that integrate diagnostic evaluation, individualized therapeutic strategies, comprehensive psychosocial support, and social dimensions. Furthermore, the management of DSD remains inherently complex and controversial regarding optimal treatment strategies, the role of genetic determinants, and the efficacy of available therapeutic modalities (3). Effectively managing these challenges requires a team-oriented approach that is directed towards medical, surgical and psychological needs (4). This includes experts in genetics, endocrinology, urology, bioethics, gynecology, and psychology. Furthermore, considerations about gender assignment, gonadal malignancy risk, hormonal suppression or replacement, fertility and several ethical, legal, and cultural dilemmas, such as irreversible surgical interventions and consent considerations, frequently arise when caring for these patients (5, 6).

In this series of articles, Frontiers of Pediatrics has addressed several areas in the diagnosis and care of patients with DSD under the research topic “*Disorders of Sex Development In Children: Advancing Multidisciplinary Approaches For Complex Diagnosis And Management.*” It explores a wide range of research in the field from the multidisciplinary care of DSD patients to the characterization of specific genetic diagnoses. These are all research areas that will educate readers, as well as encourage healthcare providers and researchers to continue the work demonstrated in published articles.

In the manuscript “*Structured care after a DSD diagnosis in childhood: a mixed methods evaluation of the Empower-DSD program*,” Wechsung et al. describe a structure pathway that was utilized to provide multidisciplinary care and information exchange in the first 8–12 weeks of patients identified with DSD. The authors show that a management program proved helpful to alleviating stress in families and developed educational materials that better equipped healthcare providers to care for these patients. This work emphasizes the importance of early access to specialized DSD teams and may help to suggest standards of care in other healthcare settings that treat patients with DSD.

Peng et al. present a more focused manuscript regarding patients with 46, XY disorders entitled, “*Profile of DHX37 gene defects in human genetic diseases: 46, XY disorders of sex development*”. The authors demonstrate knowledge gaps that exist in the genetic features and molecular mechanisms of patients with DHX37 defects. The authors characterize sixty patients with reported DHX37-related 46, XY DSD and discuss potential pathways such as ribosome synthesis and cell cycle regulation may be affected by this mutation.

In their manuscript, “*Guidance for shared decision-making regarding orchiectomy in individuals with differences of sex development due to 17-β-hydroxysteroid dehydrogenase type 3 deficiency*”, Yu et al. demonstrate the intricacies involved in decisions related to clinical management of individuals with DSD due to 17-*β*-HSD type 3 deficiency. In the last decade, there was a shift in consensus guidelines that recommends considering male gender assignment in infancy in these patients, yet the authors highlight specific considerations regarding decision-making around orchiectomy in those with 17-*β*-HSD3 deficiency, and how those can influence outcomes. They report that most individuals who had early orchiectomy and were diagnosed prior to puberty, maintained female gender identity if raised as girls. According to the authors, this suggests that age at diagnosis and timing of orchiectomy along with other factors such as sociocultural and geographic variables can affect gender identity in these individuals. Furthermore, they propose a practical tool they created called Facts, Actions, Considerations, Time (FACT) sheet that considers sex hormones, gender identity, malignancy and surgery-associated risks, fertility potential, and timing of orchiectomy to assist on decision-making regarding gonadal management.

In the manuscript of Gong et al. entitled “*Retrospective analysis of children with 46, XX testicular/ovotesticular DSD: a 10-year single-center experience*”, the authors provide a comprehensive assessment in the evaluation of patients identified with 46, XX testicular/ovotesticular differences/disorders of sexual development. In addition to phenotypic and hormonal evaluation, about 55% of patients were further evaluated with whole exome sequence. Interestingly, the authors did not find any genetic variants. The article highlights the variability of phenotypes that still occurs despite similar genotypes and the need for more rigorous genetic assessment. Moreover, the authors also describe a significant impact on the psychosocial development and identity in these patients, thus emphasizing the need for longitudinal care of these patients.

Finally, in addition to the characterizing phenotypic variability, this research topic also highlights the potential risks for neurodevelopmental compromise in some patients with DSD. The appropriate assessment and intervention can significantly improve the quality of life. Fezza et al. in their manuscript, “*Spectrum of Neuropsychological Challenges in Turner Syndrome*” provide a retrospective chart review of completed assessments in patients with Turner syndrome. They provide insight on possible genotype/phenotype correlations relating to various developmental and mental health disorders in this cohort. The importance of this data helps to further emphasizes the importance of a multidisciplinary approach that also addresses potential developmental issues. Finally, it offers anticipatory guidance regarding timely intervention for providers of patients diagnosed with Turner syndrome.

In summary, this research topic addressing the field of DSD provides just a glimpse at the advances in diagnostic and clinical care of DSD patients. Yet, despite the advances in knowledge and the improvements in genetic evaluation, the presentation of DSD continues to be accompanied by some uncertainty and confusion for not just parents, but providers as well. Therefore, it is essential that care providers and scientists from all aspects in the care of children with DSD remain vigilant in the efforts of education, clinical care and research in the field of DSD.

## References

[1] Lee PA Nordenstrom A Houk CP Ahmed SF Auchus R Baratz A Global disorders of sex development update since 2006: perceptions, approach and care. Horm Res Paediatr. (2016) 85(3):158–80. 10.1159/000442975 26820577

[2] León NY Reyes AP Harley VR. A clinical algorithm to diagnose differences of sex development. Lancet Diabetes Endocrinol. (2019) 7(7):560–74. 10.1016/S2213-8587(18)30339-5 30803928

[3] Lee PA Fuqua JS Houk CP Kogan BA Mazur T Caldamone A. Individualized care for patients with intersex (disorders/differences of sex development): part I. J Pediatr Urol. (2020) 16(2):230–7. 10.1016/j.jpurol.2020.02.013 32249189

[4] Nowotny HF Reisch N. Challenges waiting for an adult with DSD. Horm Res Paediatr. (2023) 96(2):207–21. 10.1159/000527433 36473446

[5] Johnson EK Whitehead J Cheng EY. Differences of sex development: current issues and controversies. Urol Clin North Am. (2023) 50(3):433–46. 10.1016/j.ucl.2023.04.010 37385705

[6] Sandberg DE Gardner M. Differences/disorders of sex development: medical conditions at the intersection of sex and gender. Annu Rev Clin Psychol. (2022) 18:201–31. 10.1146/annurev-clinpsy-081219-101412 35216524 PMC10170864

